# Time fractional analysis of channel flow of couple stress Casson fluid using Fick’s and Fourier’s Laws

**DOI:** 10.1038/s41598-022-06897-y

**Published:** 2022-02-22

**Authors:** Shafiq Ahmad, Sami Ul Haq, Farhad Ali, Ilyas Khan, Kottakkaran Sooppy Nisar

**Affiliations:** 1 Department of Mathematics, Islamia College Peshawar, 25000 Peshawar, Pakistan; 2 Department of Mathematics, City University of Science and Information Technology, Peshawar, 25000 Pakistan; 3 Department of Mathematics, College of Science Al-Zulfi, Majmaah University, Al Majma’ah, 11952 Saudi Arabia; 4 Department of Mathematics, College of Arts and Science, Prince Sattam Bin Abdulaziz University, Wadi Al-Dawasir, 11991 Saudi Arabia

**Keywords:** Applied mathematics, Computational science

## Abstract

This study aim to examine the channel flow of a couple stress Casson fluid. The flow is generated due to the motion of the plate at $y=0$, while the plate at $y=d$ is at rest. This physical phenomenon is derived in terms of partial differential equations. The subjected governing PDE’s are non-dimensionalized with the help of dimensionless variables. The dimensionless classical model is generalized by transforming it to the time fractional model using Fick’s and Fourier’s Laws. The general fractional model is solved by applying the Laplace and Fourier integral transformation. Furthermore, the parametric influence of various physical parameters like Casson parameter, couple stress parameter, Grashof number, Schmidt number and Prandtl number on velocity, temperature, and concentration distributions is shown graphically and discussed. The heat transfer rate, skin friction, and Sherwood number are calculated and presented in tabular form. It is worth noting that the increasing values of the couple stress parameter $\lambda$ deaccelerate the velocity of Couple stress Casson fluid.

## Introduction

Fractional calculus has been developing immensely these days due to its flexible and eccentric properties. Fractional calculus is the expansion of classical calculus, and it has around three centuries-old histories. This type of derivatives is an important tool for classifying various systems such as memory. Fractional calculus has numerous applications in many fields which includes Bio-Maths^1–3^, waves analysis^4^, diffusion^5–8^, Gas and smoking dynamics^9,10^ and transportation of water in ground level^11^. Awan et al.^12^ developed new fractional estimates for Hermite–Hadamard’s inequalities using Riemann–Liouville fractional integrals. Jumarie^13^ proposed a modified Riemann–Liouville definition to find Taylor’s expansion of fractional order for the functions that are not differentiable. In another paper, the same author Jumarie^14^ defined fractional Laplace transform based on modified Riemann Liouville derivative. Owolabi^15^ discussed the stability of Chaotic differential equations with the help of Riemann–Liouville fractional derivative. Refai et al.^16^ analyzed one-dimensional fractional diffusion initial-boundary-value problems with the help of the Riemann–Liouville time-fractional derivative. Mohamed et al.^17^ developed the approximate solutions of the cubic nonlinear fractional Schordinger equation using Adomian decomposition method by introducing Caputo fractional derivatives. Using Caputo fractional derivatives, Zhao et al.^18^ established new inequalities such as Hermite Jensen Mercer type inequalities. Baleanu^19^ studied discrete constrained systems and presented the fractional dynamics for the said system using Caputo derivatives. In^20^, Atangana and Baleanu developed the Mittag Leffler function in 2016, which is very useful in finding the solution of fractional order integral equation or fractional order differential equations. Sheikh et al.^21^ investigated the exact solutions for the flow of Casson fluid with the help of Atangana–Baleanu fractional derivatives. Atangana–Baleanu fractional derivatives approach was utilized by Nadeem et al.^22^ to enhance the proficiency of solar plates. They used a slanted plane to understand the better performance of the solar plates. Ramanaiah^23^ shown that by taking CS fluid as a lubricant, the squeeze time can be increased.

The heat transfer includes more complicated operations like metabolic heat generation and warm change due to numerous exogenous connections. Heat transfer can be used in a variety of applications, including non-Newtonian liquids, and is particularly useful in blood rheology. Taylor et al.^24^ discussed the impact of heat transfer research on US economy. Thermal therapy is a technique used to kill cancer cells^25^. The combined effect of heat and mass transfer in the nanofluid flow has been discussed by Vajjha and Das^26^. Hassnain et al.^27^ considered the combined impact of heat and mass distribution on the unsteady flow of the boundary layer. They have also considered the Newtonian heating effect. Idowu and Falodun^28^ discussed heat and mass transfer processes on Walters-B viscoelastic and Casson fluids.

Casson Fluid is one of the non-Newtonian fluid^29^. A few of the Casson liquid comprises of ketchup, molten polymers, blood, jelly, toothpaste, etc. Casson fluids are widely used in paints, drilling, metallurgy, china clay, food processing, synthetic lubricants, and bioengineering operations^30,31^. Ismael^32^ investigated the impact of energy transfer with the internal heat source on Carreau-Casson Fluids flow and used the numerical scheme $RK-4$ method to find the numerical solution of governing equations. Raju et al.^33^ developed a mathematical model of Casson and Williamson fluid flow to investigate the flow, heat, and mass transfer over a stretching surface. Loganathan and Deepa^34^ investigated the flow of convective Casson fluid paste a Riga plate. Rafiq^35^ studied Casson fluid flow generated by the non-coaxial rotation of a disk. Exact solutions of BVP are obtained by using Laplace transform technique. Model et al.^36^ used variational principle to optimize the total stress of the casson and Ree-Eyring fluids flow. Nadeem et al.^37^ use fractional derivatives to develop the generalized Casson fluid model. Hussain et al.^38^ discussed the behavior of Casson fluid flow under the influence of magnetic field and obtained the numerical solutions using shooting method of the involved equations.

Applications of CSF are discussed in^39,40^. Tripathi et al.^41^, used MHD flow of a CS Nanofluid over a convective wall have discussed. Reddy et al.^42^ studied the hydromagnetic peristaltic motion of CS fluid in a slanted channel keeping the wavelength long and Reynolds number small. Farooq et al.^43^ discussed plane Poiseuille flow, Couette flow, generalized Couette flow and, plug flow of variable viscosity of Couple stress fluid. Alsaedi^44^, used Homotopy technique for the solution of CS fluid flow of a melting heat transfer problem. They concluded that by increasing CS parameter, the boundary layer thickness decreases, while the surface heat transfer and temperature increase. Akhtar^45^, Used Caputo and Caputo-Fabrizio time-fractional derivatives definition to find the solutions of the CS fluid channel flow and then compare the results. Stokes^46^, discussed the impact of Couples stresses in fluids by considering different BVP. Stokes^47^ shown that for Poiseuille flow, the impact of Couple stresses are immense for large Hartmann numbers whenever the magnetic field is applied across the plates. Arif et al.^48^ used the Caputo-Fabrizio derivatives to calculate the solution of CS fluid generalized Couette flow. The same authors in another paper^49^ presented the solutions of CS fluid in parallel plates for heat transfer. Recently, Arif et al.^50^ used the Atangana Baleanu definition of fractional derivatives to develop the closed form solutions of couple stress nanofluids flow in a channel. Most recently, Ali et al.^51^, considered the flow of laminar and unsteady couple stress fluid between infinite plates. They considered engine oil as a base fluid and exact solutions are obtained by using Laplace and Fourier transforms. They concluded that the engine oil efficiency has been improved by 12.8 percent by adding nanoparticles.

In the above literature no one has considered couple stress Casson fluid in a channel using Fick’s and Fourier’s laws to find the closed form solutions. In this article, we considered the flow of couple stress Casson fluid in a channel and the flow is generated due to the motion of the plate at $y=0$. The governing PDE’s are non-dimensionalized using suitable dimensionless variables and the energy and mass equations are transformed to fractional model using Fick’s and Fourier’s Laws. Caputo definition is used for the fractional model and then these fractional PDE’s are solved by using the joint application of Laplace and Fourier transforms. The obtained results are depicted in the form of figures. The impact of different parameters on Nusselt and Sherwood numbers and skin fraction are shown in tables.

## Mathematical formulation

**Figure 1:**
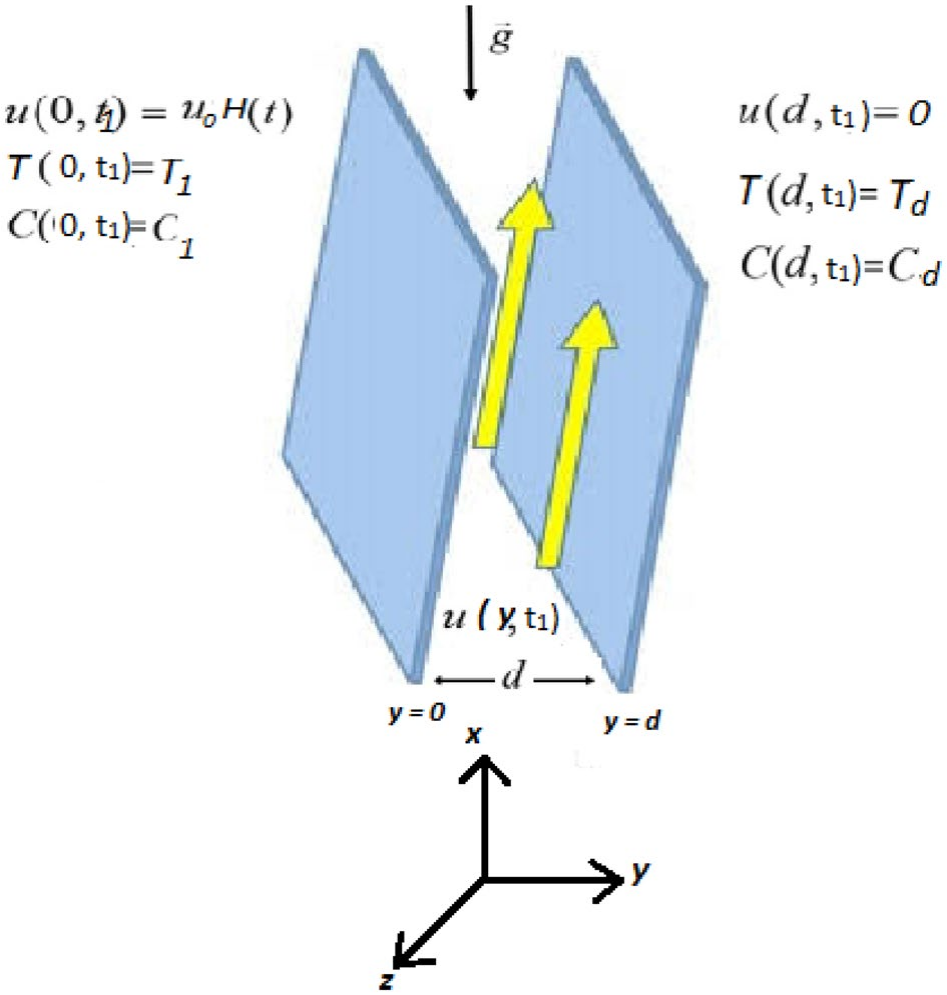
Geometry of the problem.

Consider the motion of couple stress Casson fluid between in a infinite parallel plates.The flow is considered in x direction. Initially, $(t\le 0)$ the fluid as well as both the plates are at rest having ambient temperature $T_{d}$ and concentration $C_d$. After some time *t* $(t>0)$, the plate at $y=0$ is dragged with constant velocity $u_0H(t)$ where $u_0$ is the characteristic velocity and second plate remains a rest. The temperature and concentration of the moving plate raise to $T_1$ and $C_1$ respectively and then remains constant as shown in the Fig. 1.

For the velocity $v=(u(y,t), 0, 0)$, the equation of continuity is identically satisfied and using the well known Boussinesq’s approximation, the governing equations for unsteady couple stress Casson fluid flow between infinite parallel plates^52^ are given by:

The momentum equation is:

1 $$\begin{aligned} \rho \frac{\partial u}{\partial t}=\mu \left( 1 +\frac{1}{\beta }\right) \frac{\partial ^2u}{\partial y^2} -\eta \frac{\partial ^4u}{\partial y^4} +\vec {g}(\rho B_{T})(\hbox {T}\!\!\!\hbox {-}-\hbox {T}\!\!\!\hbox {-}_{d}) +g(\rho B_{c})(C-C_{d}). \end{aligned}$$

2 $$\begin{aligned} (\rho C_{p}) \frac{\partial T(y,t)}{\partial t} = -\frac{\partial q(y,t)}{\partial y}. \end{aligned}$$

The Fourier’s law:

3 $$\begin{aligned} q(y,t) = -k\frac{\partial \hbox {T}\!\!\!\hbox {-}(y,t)}{\partial y}. \end{aligned}$$

The Thermal balance equation:

4 $$\begin{aligned} \frac{\partial C(y,t)}{\partial t} = -\frac{\partial S(y,t)}{\partial y}. \end{aligned}$$

The Fick’s law:

5 $$\begin{aligned} S(y,t) = -{D} \frac{\partial C(y,t)}{\partial y}. \end{aligned}$$

The initial and boundary conditions are:

6 $$\begin{aligned} \begin{aligned} u(y,0) & =0, \quad u(0,t)=u_{0} H(t), \quad u(d,t)=0,\\ \hbox {T}\!\!\!\hbox {-}(y,0) & =\hbox {T}\!\!\!\hbox {-}_d, \quad \hbox {T}\!\!\!\hbox {-}(0,t)=\hbox {T}\!\!\!\hbox {-}_{1},\quad \hbox {T}\!\!\!\hbox {-}(d,t)=\hbox {T}\!\!\!\hbox {-}_d\\ C(y,0) & =C_d, \quad C(0,t)=C_{1} ,\quad C(d,t)=C_d. \end{aligned} \end{aligned}$$

Here u, $\hbox {T}\!\!\!\hbox {-}$ and C are the velocity, temperature and concentration of the fluid respectively. $\rho$, $(\rho C{p})$, $\mu$, $\eta$, *K*, , *g*, and *D* is the density, specific heat,  Dynamic viscosity, couple stress parameter, thermal conductivity, gravitational acceleration and mass diffusivity respectively. To obtain Dimensionless system of PDE’s, we Introduce the following dimensionless variables:

7 $$\begin{aligned} \begin{aligned} y^{*}=\frac{y}{d},\,\,\,\, \quad u^{*}=\frac{u}{v_{0}}, \,\,\,\, \quad t^{*}=\frac{\nu t}{d^2},\,\,\,\,\, \quad \hbox {T}\!\!\!\hbox {-}^{*} = \frac{\hbox {T}\!\!\!\hbox {-}-\hbox {T}\!\!\!\hbox {-}_{d}}{\hbox {T}\!\!\!\hbox {-}_{1}-\hbox {T}\!\!\!\hbox {-}_{d}},\\ C^*= \frac{C-C_{d}}{C_{1}-C_{d}}, \quad q^{*}=\frac{qd}{k(T_{1}-T_{d})}, \quad s^{*}=\frac{sd}{D(C_{1}-C_{d})}.\ \end{aligned} \end{aligned}$$

By using these dimensionless variables and dropping the $*$ signs, the Eqs. (1–8) becomes:

8 $$\begin{aligned} \frac{\partial u(y,t)}{\partial t}=\left( 1 +\frac{1}{\beta }\right) \frac{\partial ^2u(y,t)}{\partial y^2} -\lambda \frac{\partial ^4u(y,t)}{\partial y^4} +G_r\hbox {T}\!\!\!\hbox {-}(y,t) +G_mC(y,t). \end{aligned}$$

9 $$\begin{aligned} \frac{\partial \hbox {T}\!\!\!\hbox {-}(y,t)}{\partial t} = -\frac{1}{Pr}\frac{\partial q(y,t)}{\partial y}. \end{aligned}$$

10 $$\begin{aligned} q(y,t) =-\frac{\partial \hbox {T}\!\!\!\hbox {-}(y,t)}{\partial y}. \end{aligned}$$

11 $$\begin{aligned} \frac{\partial C(y,t)}{\partial t} = -\frac{1}{Sc}\frac{\partial S(y,t)}{\partial y}. \end{aligned}$$

12 $$\begin{aligned} S(y,t) = -\frac{\partial C(y,t)}{\partial y}. \end{aligned}$$

13 $$\begin{aligned} \begin{aligned} u(y,0) & =0, \quad u(0,t)=1, \quad u(d,t)=0,\\ \hbox {T}\!\!\!\hbox {-}(y,0) & =0, \quad \hbox {T}\!\!\!\hbox {-}(0,t)={1}, \quad \hbox {T}\!\!\!\hbox {-}(d,t)=0,\\ C(y,0) & =0, \quad C(0,t)={1} , \quad C(d,t)=0,\\ \frac{\partial ^2u(0,t)}{\partial y^2} & =\frac{\partial ^2u(d,t)}{\partial y^2}=0. \end{aligned} \end{aligned}$$

where *Pr*, *Sc*, *Re*, *Gr*, *Gm* and $\lambda$ are the Prandtl number, Schmidt number, Reynolds number, thermal and mass Grashof numbers and non-dimensional couple stress parameter.

The Generalized Fourier’s and Fick’s are used as under:

14 $$\begin{aligned} q(y,t) =-^{C}D_{t}^{1-\alpha }\left( \frac{\partial \hbox {T}\!\!\!\hbox {-}(y,t)}{\partial y}\right) ; \quad 0<\alpha \le 1. \end{aligned}$$

15 $$\begin{aligned} S(y,t) = -^{C}D_{t}^{1-\alpha }\left( \frac{\partial C(y,t)}{\partial y}\right) ; \quad 0<\alpha \le 1. \end{aligned}$$

where $^{C}D_{t}^{1-\alpha }(.)$ is the Caputo time Fractional Operator and is defined as;

16 $$\begin{aligned} ^{C}D_{t}^{\alpha }(K(y,t))=\frac{1}{\Gamma (1-\alpha )}\int _{0}^{t}K^{.}(y,s)(t-s)^{-\alpha }ds =\zeta _\alpha (t)*K(y,t). \end{aligned}$$

Here $\zeta _\alpha (t)=\frac{t^{-\alpha }}{\Gamma (1-\alpha )}$ is the singular power law kernel. Futhermore,

17 $$\begin{aligned} L(\zeta _\alpha (t))=\frac{1}{s^{1-\alpha }}, \,\,\,\left( \zeta _{1-\alpha }*\zeta _\alpha \right) (t)=1, \,\,\,\zeta _0(t)=L^{-1}\left( \frac{1}{s}\right) =1,\,\,\,\zeta _1(t)=L^{-1}(1)=\delta (t). \end{aligned}$$

Here $\delta (t)$ is the Dirac’s delta function. Using Eq. (16) and the above properties given in (17), we can write:

18 $$\begin{aligned} \begin{aligned} ^{C}D_{t}^{0}(K(y,t))&=K(y,t)-K(y,0).\\ ^{C}D_{t}^{1}(K(y,t))&=\frac{\partial C(y,t)}{\partial t}. \end{aligned} \end{aligned}$$

Using the definition of Caputo fractional operator in equation (16), and also from Eqs. (9, 11, 14, 15), we can write:

19 $$\begin{aligned} \frac{\partial \hbox {T}\!\!\!\hbox {-}(y,t)}{\partial y}=\frac{1}{Pr}^{C}D_{t}^{1-\alpha }\left( \frac{\partial ^2 \hbox {T}\!\!\!\hbox {-}(y,t)}{\partial y^2}\right) . \end{aligned}$$

20 $$\begin{aligned} \frac{\partial C(y,t)}{\partial y}=\frac{1}{Sc}^{C}D_{t}^{1-\alpha }\left( \frac{\partial ^2 C(y,t)}{\partial y^2}\right) . \end{aligned}$$

To find the simplify form of Eqs. (19) and (20), we consider the time fractional integral operator:

21 $$\begin{aligned} \nu _{t}^{\alpha }(K(y,t))=\left( \zeta _{1-\alpha }*K\right) (t)=\frac{1}{\Gamma (\alpha )}\int _{0}^{t}K(y,s)(t-s)^{\alpha -1}ds. \end{aligned}$$

The Eq. (21) is the inverse operator of the fractional derivative $^CD^\alpha _t$ defined in Eq. (16). Using the properties defined in^52^, the Eqs. (19) and (20) can be written as:

22 $$\begin{aligned} ^{C}D_{t}^{\alpha }{\hbox {T}\!\!\!\hbox {-}}(y,t)=\frac{1}{Pr}\frac{\partial ^2{\hbox {T}\!\!\!\hbox {-}}(y,t)}{\partial y^2}. \end{aligned}$$

23 $$\begin{aligned} ^{C}D_{t}^{\alpha }C(y,t)=\frac{1}{Sc}\frac{\partial ^2{C}(y,t)}{\partial y^2}. \end{aligned}$$

## Solution of the problem

### Solution of energy field

Using Laplace transform technique (LT) to Eq. (22), we have:

24 $$\begin{aligned} s^{\alpha } \bar{{\hbox {T}\!\!\!\hbox {-}}}(y,s) = \frac{1}{Pr}\frac{d^{2} \bar{{\hbox {T}\!\!\!\hbox {-}}}(y,s)}{d y^2}. \end{aligned}$$

and the transformed initial and boundary conditions are given by:

25 $$\begin{aligned} \begin{aligned} {\bar{u}}(y,0) & =0, \quad {\bar{v}}(0,s) = \frac{1}{s}, \quad {\bar{u}}(1,s) = 0, \\ \bar{{\hbox {T}\!\!\!\hbox {-}}}(y,0) & =0, \quad \bar{{\hbox {T}\!\!\!\hbox {-}}}(0,s) = \frac{1}{s}, \quad \bar{{\hbox {T}\!\!\!\hbox {-}}}(1,s) = 0, \\ {\bar{C}}(y,0) & =0, \quad {\bar{C}}(0,s) = \frac{1}{s}, \quad {\bar{C}}(1,s) = 0. \end{aligned} \end{aligned}$$

Now applying the Finite Sine Fourier Transform to Eq. (24) and using the conditions in Eq. (25), we obtain:

26 $$\begin{aligned} \tilde{\bar{{\hbox {T}\!\!\!\hbox {-}}}}(k,s) = \frac{ k \pi }{Pr}. \frac{1}{s[s^{\alpha } + L]}. \end{aligned}$$

where $$L=\frac{(k \pi )^2}{Pr}$$.

Applying Inverse Transformations, Eq. (26) takes the following shape:

27 $$\begin{aligned} {\hbox {T}\!\!\!\hbox {-}}(y,t) = (1- y) - 2\sum _{k=1}^{\infty } \frac{1}{k \pi }. E_{\alpha } \left[ \frac{-(k\pi )^{2} t^{\alpha }}{Pr}\right] sin(k\pi y). \end{aligned}$$

### Solution of concentration field

Using Laplace transform technique (LT) to Eq. (23), we get:

28 $$\begin{aligned} s^{\alpha } {\bar{C}}(y,s) = \frac{1}{Sc}\frac{d^{2} {\bar{C}}(y,s)}{d y^2}. \end{aligned}$$

Now applying the Finite Sine Fourier Transform to Eq. (28) and using the conditions in Eq. (25), we can write:

29 $$\begin{aligned} \tilde{{\bar{C}}}(k,s) = \frac{ k \pi }{Sc}. \frac{1}{s[s^{\alpha } + L']}. \end{aligned}$$

where $L'=\frac{(k \pi )^2}{Sc}$. Applying Inverse Transformation to Eq. (29),we have:

30 $$\begin{aligned} C(y,t) = (1- y) - 2\sum _{k=1}^{\infty } \frac{1}{k \pi }. E_{\alpha } \left[ \frac{-(k\pi )^{2} t^{\alpha }}{Pr}\right] . sin(k\pi y). \end{aligned}$$

where $E_\alpha \left( -at^{\alpha }\right) = \sum _{k=0}^{\infty }\frac{\left( -at\right) ^{k}}{\Gamma (\alpha k+1)}$ is known as MITTAG-LEFFLER Function.

### Solution of momentum equation

Using Laplace transform to Eq. (8), we can write:

31 $$\begin{aligned} {\bar{u}}(y,s)=\left( 1 +\frac{1}{\beta }\right) \frac{\partial ^2{\bar{u}}(y,s)}{\partial y^2} -\lambda \frac{\partial ^4{\bar{u}}(y,s)}{\partial y^4} +G_r\bar{{\hbox {T}\!\!\!\hbox {-}}}(y,s) +G_m{{\bar{C}}}(y,s). \end{aligned}$$

Now applying Finite Fourier Sine Transform to Eqs. (31) and substituting Eqs.(26) and (29), we have:

32 $$\begin{aligned} \begin{aligned} {\bar{u}}(k,s)&=\frac{R_1}{R_2s}-\frac{R_1}{R_2(s+R_2)} +\frac{G_rK\pi }{PrR_2}\left( \frac{1}{s(s^\alpha +L)}-\frac{1}{(s+R_2)(s^\alpha +L)}\right) \\&\quad +\frac{G_m K \pi }{ScR_2}\left( \frac{1}{s(s^\alpha +L^{'})}-\frac{1}{(s+R_2)(s^\alpha +L^{'})}\right) . \end{aligned} \end{aligned}$$

Taking Inverse Laplace Transform, Eq. (32) can be written as:

33 $$\begin{aligned} \begin{aligned} {\tilde{u}}(k,t)&=\frac{R_1}{R_2}\left( 1-e^{-R_2t}\right) +\frac{G_r}{k \pi }\left[ 1-E_\alpha \left( -\frac{(k \pi )^2}{Pr}t^\alpha \right) \right] +\frac{G_m}{k \pi }\left[ 1-E_\alpha \left( -\frac{(k \pi )^2}{Sc}t^\alpha \right) \right] \\&\quad -\frac{G_rK\pi }{PrR_2}\int _{0}^{\tau }t^{\alpha -1}E_{\alpha ,\alpha }\left( -Lt^\alpha \right) *e^{-R_2(t-\tau )}d\tau -\frac{G_mK\pi }{ScR_2}\int _{0}^{\tau }t^{\alpha -1}E_{\alpha ,\alpha }\left( -L^{'}t^\alpha \right) *e^{-R_2(t-\tau )}d\tau . \end{aligned} \end{aligned}$$

Now by taking Inverse Fourier Finite Sine transformation of Eq. (33) , the final exact solution of the Eq. (31) is:

34 $$\begin{aligned} \begin{aligned} u(y,t)&=1-y-2\sum _{k=1}^{\infty }\frac{1}{k \pi }e^{-k \pi R_2 t}sin\left( k \pi y\right) + 2G_r \sum _{k=1}^{\infty }\frac{1}{k \pi }\left[ 1-E_\alpha \left( -\frac{(k \pi )^2}{Pr}t^\alpha \right) \right] sin(k\pi y)\\&\quad + 2G_m \sum _{k=1}^{\infty }\frac{1}{k \pi }\left[ 1-E_\alpha \left( -\frac{(k \pi )^2}{Sc}t^\alpha \right) \right] sin(k\pi y) -2\frac{G_r}{Pr}\sum _{k=1}^{\infty }\frac{k\pi }{R_2}sin(k\pi y)\int _{0}^{\tau }t^{\alpha -1}E_{\alpha ,\alpha }\left( -Lt^\alpha \right) \times e^{-R_2(t-\tau )}d\tau \\&\quad -2\frac{G_m}{Sc}\sum _{k=1}^{\infty }\frac{k\pi }{R_2}sin(k\pi y)\times \int _{0}^{\tau }t^{\alpha -1}E_{\alpha ,\alpha }\left( -L^{'}t^\alpha \right) *e^{-R_2(t-\tau )}d\tau . \end{aligned} \end{aligned}$$

where $$R=\left( 1+\frac{1}{\beta }\right)$$, $$R_1=R(k \pi )+\lambda (k \pi )^3$$ and $$R_2=K \pi R_1$$.

## Skin friction, Nusselt and Sherwood number

### Skin friction

The skin friction is the Friction between a moving fluid and its surface enclosure. Mathematical form of the Skin friction Couple stress Casson flow in dimension form as:

35 $$\begin{aligned} Sf(0,t)=\mu \left( 1+\frac{1}{\beta }\right) \frac{\partial u}{\partial y}|_{y=0}-\eta \frac{\partial ^3 u}{{\partial y}^3}|_{y=0}. \end{aligned}$$

Introducing the dimensionless parameter, the Non-Dimensional form of the skin friction defined in Eq. (35) can be stated as:

36 $$\begin{aligned} Sf(0,t)=\left( 1+\frac{1}{\beta }\right) \frac{\partial u}{\partial y}|_{y=0}-\lambda \frac{\partial ^3 u}{{\partial y}^3}|_{y=0}. \end{aligned}$$

### Nusselt number

The ratio of convective to conductive heat transfer around a boundary is the Nusselt number.

The mathematical form of Nusselt number for Couple stress Casson fluid is defined as:

37 $$\begin{aligned} Nu=\frac{\partial \hbox {T}\!\!\!\hbox {-}}{\partial y}|_{y=0}. \end{aligned}$$

### Sherwood number

The mathematical form of Sherwood Number for Couple stress Casson fluid is defined as:

38 $$\begin{aligned} Sh=\frac{\partial C}{\partial y}|_{y=0}. \end{aligned}$$

## Results and discussion

**Figure 2:**
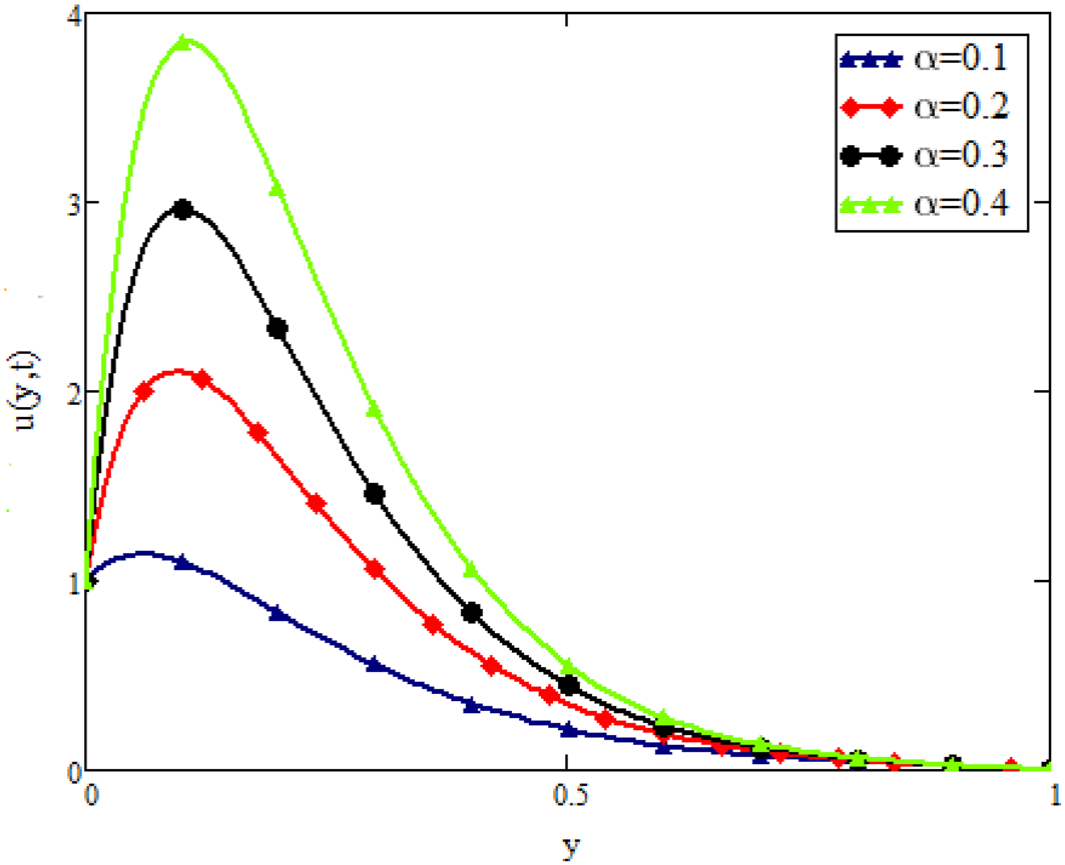
Impact of $\alpha$ on velocity distribution when $t=1$, $Gr=1.5$, $Gm=1.5$, $Pr=50$, $Sc=20$, and $\beta = 2$.

**Figure 3:**
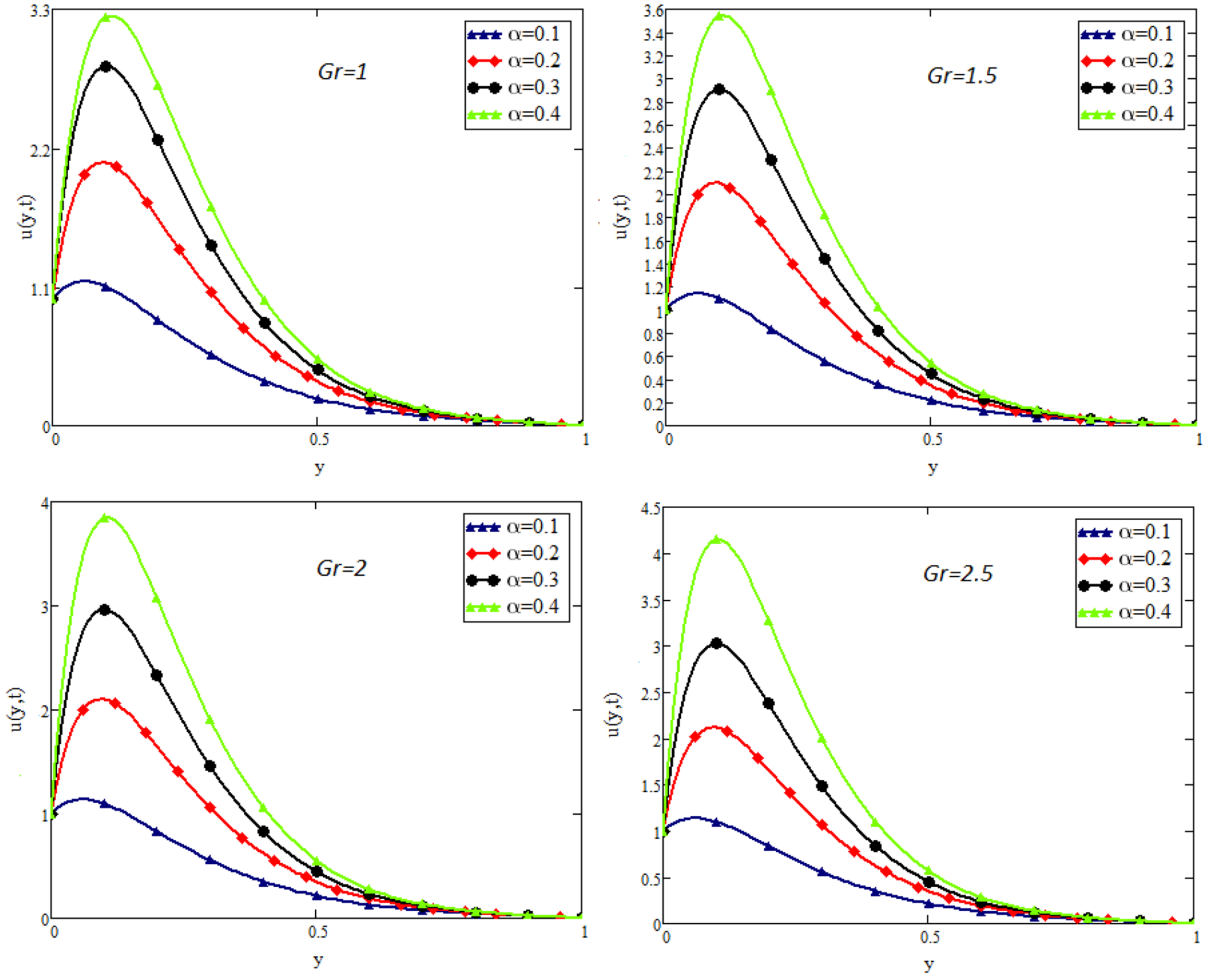
Impact of *Gr* on velocity distribution when $t=1$, $Gm=1.5$, $Pr=50$, $Sc=20$, $\beta = 2$, and $\lambda =1$.

**Figure 4:**
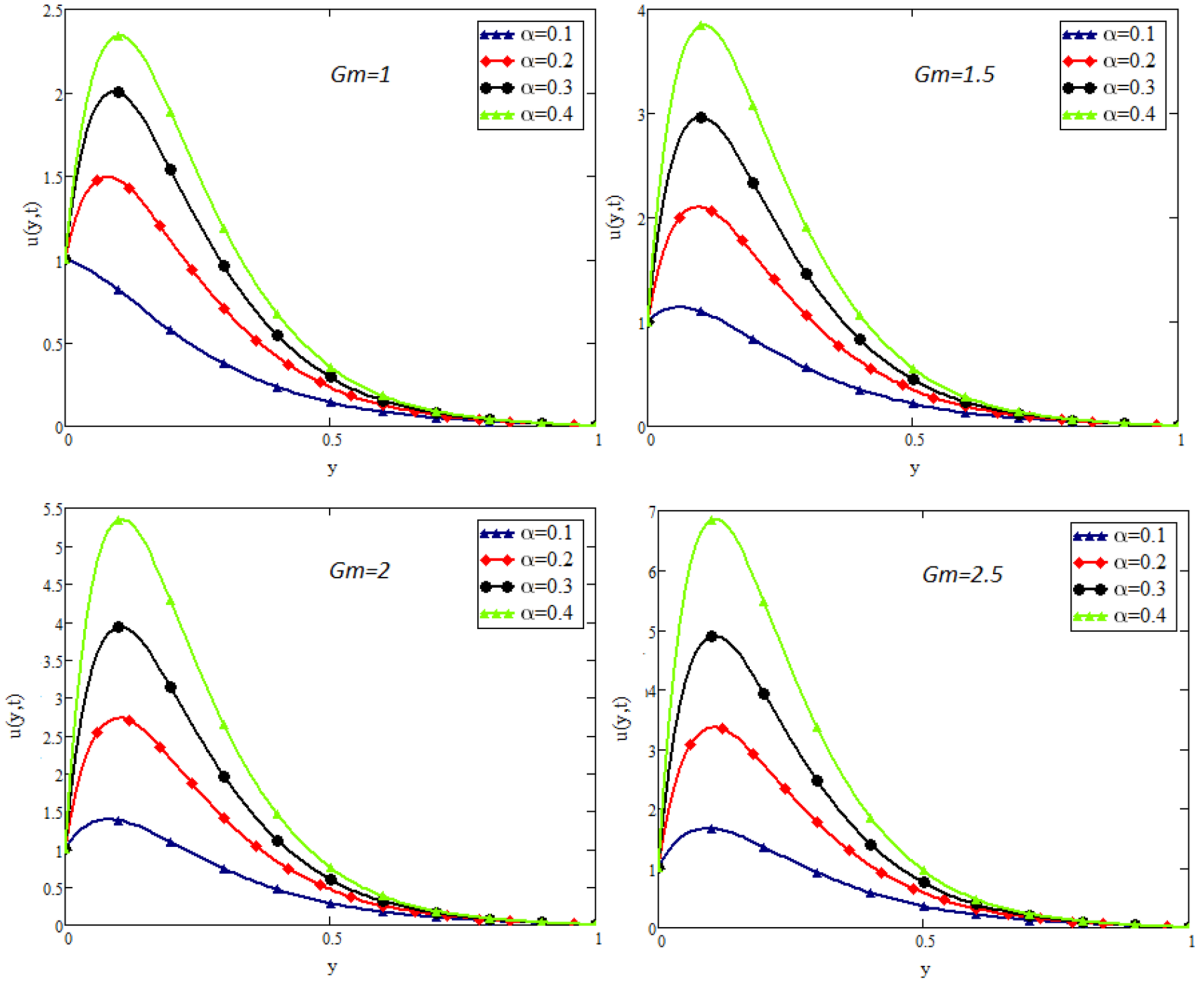
Impact of *Gm* on velocity distribution when $t=1$, $Gr=1.5$, $Pr=50$, $Sc=20$, $\beta = 2$, and $\lambda =1$.

**Figure 5:**
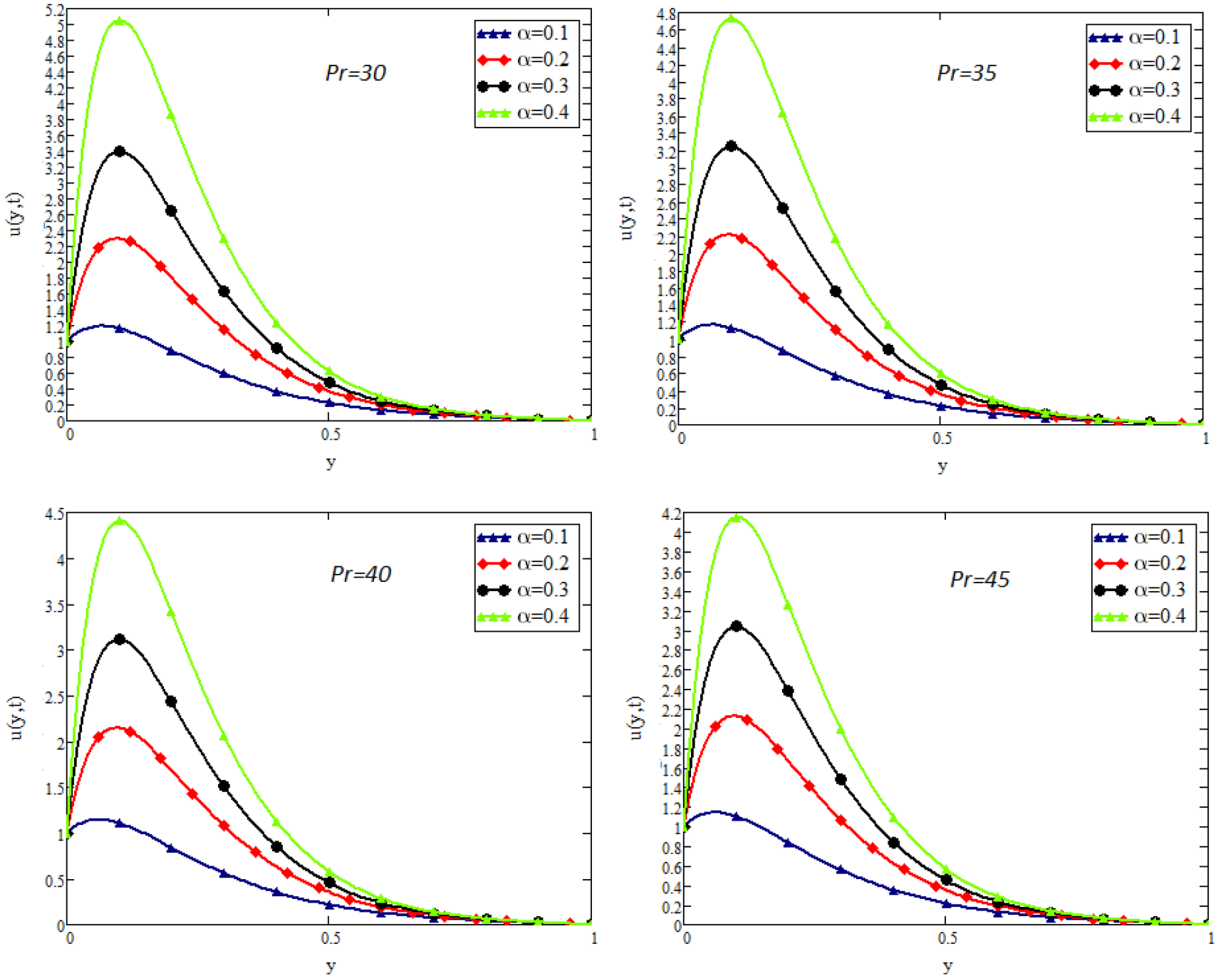
Impact of *Pr* on velocity distribution when $t=1$, $Gr=1.5$, $Gm=1.5$, $Sc=20$, $\beta = 2$, and $\lambda =1$.

**Figure 6:**
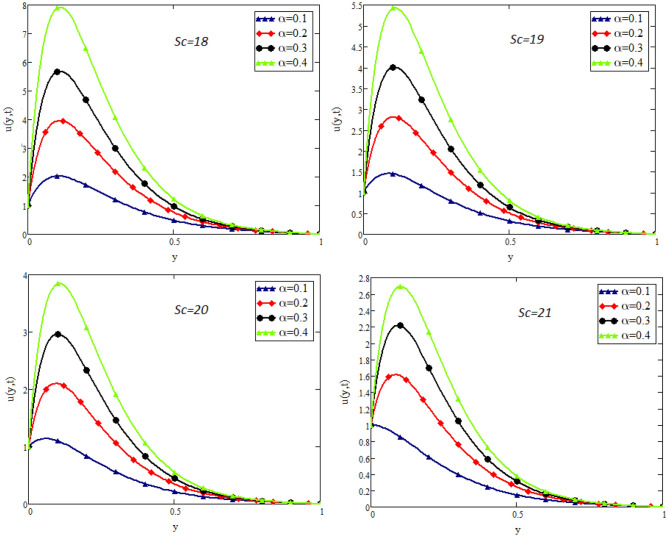
Impact of *Sc* on velocity distribution $t=1$, $Gr=1.5$, $Gm=1.5$, $Pr=50$, $\beta = 2$, and $\lambda =1$.

**Figure 7:**
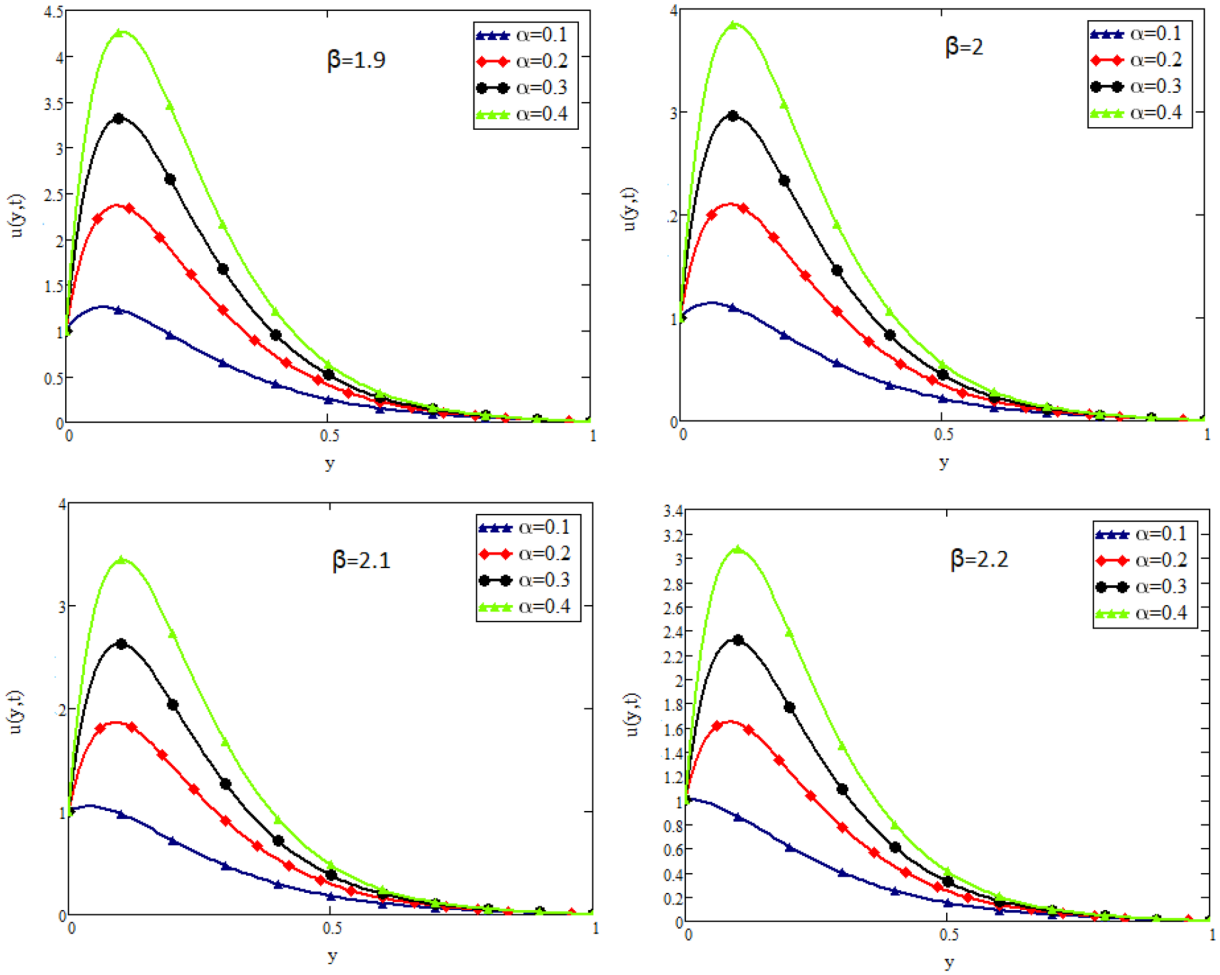
Impact of $\beta$ on velocity distribution when $t=1$, $Gr=1.5$, $Gm=1.5$, $Pr=20$, $Sc=20$, and $\lambda =1$.

**Figure 8:**
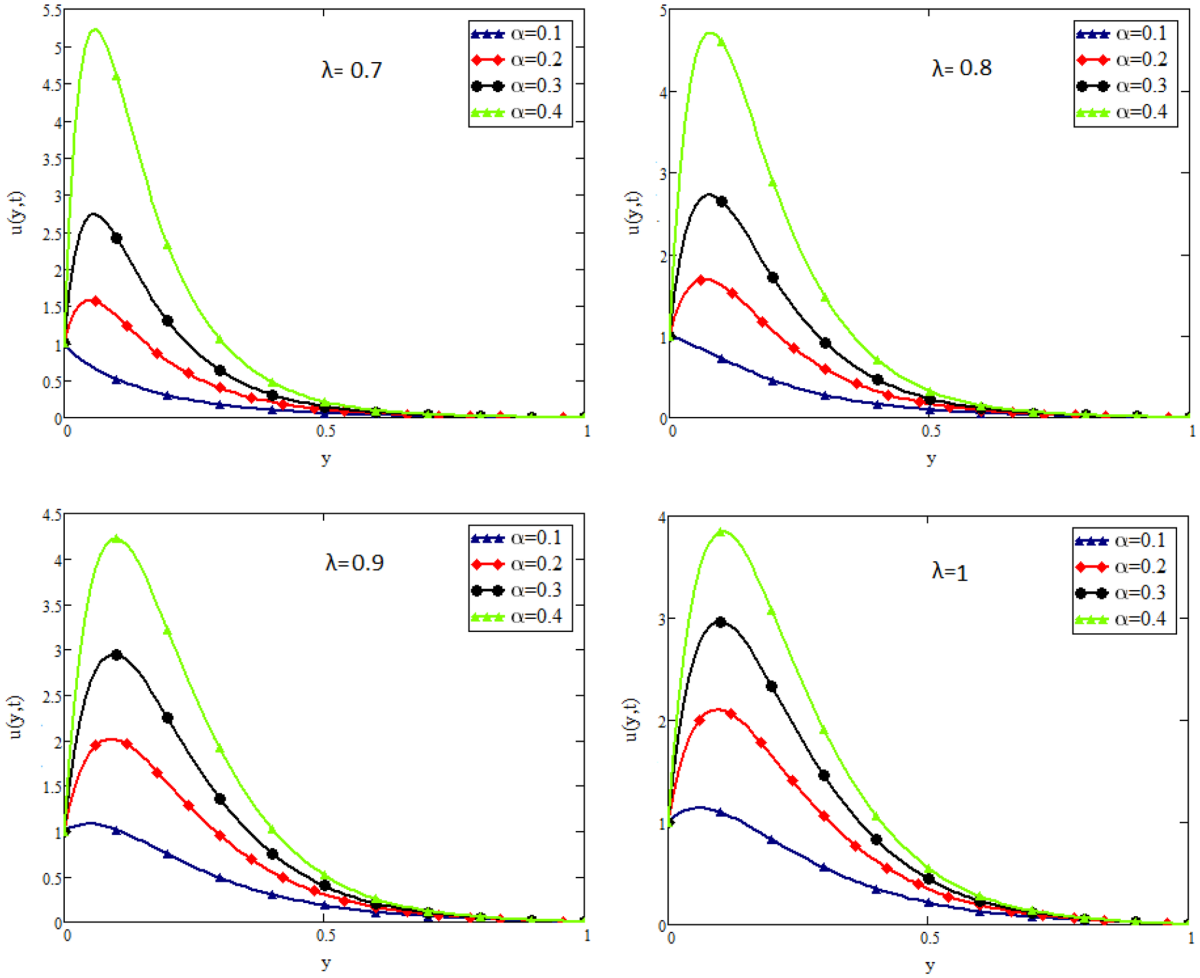
Impact of $\lambda$ on velocity distribution when $t=1$, $Gr=1.5$, $Gm=1.5$, $Pr=20$, $Sc=20$, and, $\beta =2$.

**Figure 9:**
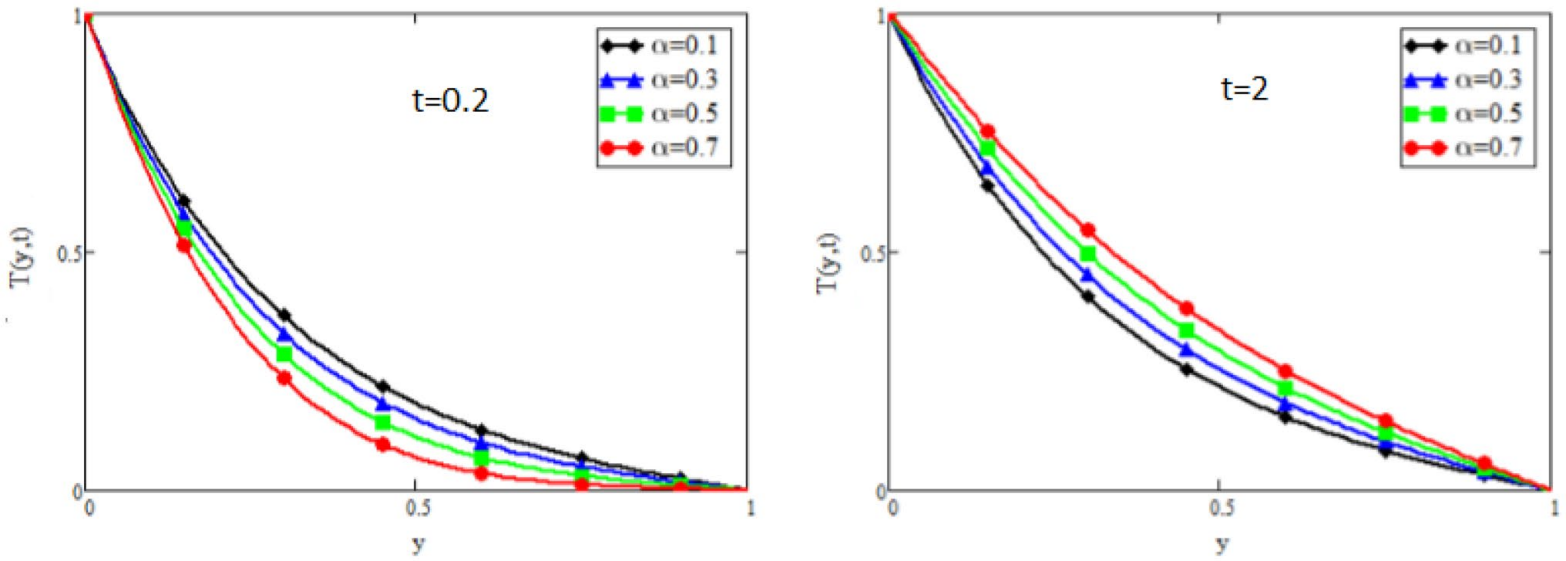
Impact of $\alpha$ on temperature distribution when $Pr=50$.

**Figure 10:**
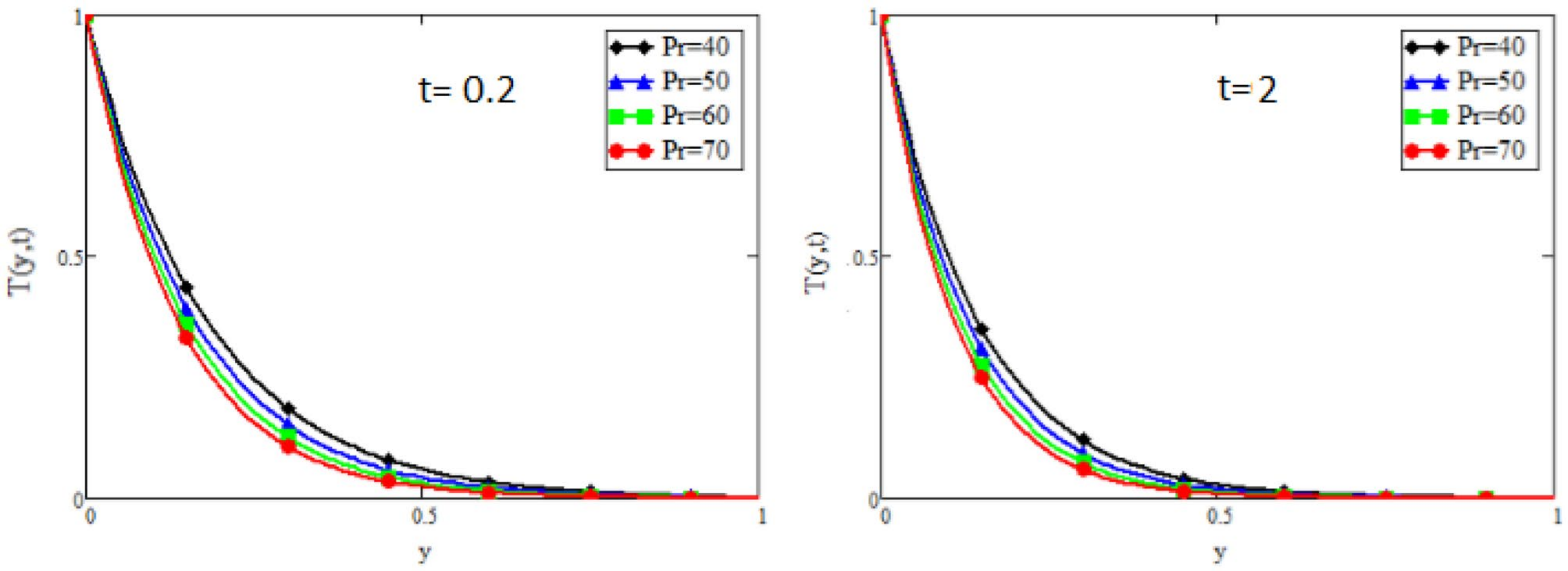
Impact of *Pr* on temperature distribution.

**Figure 11:**
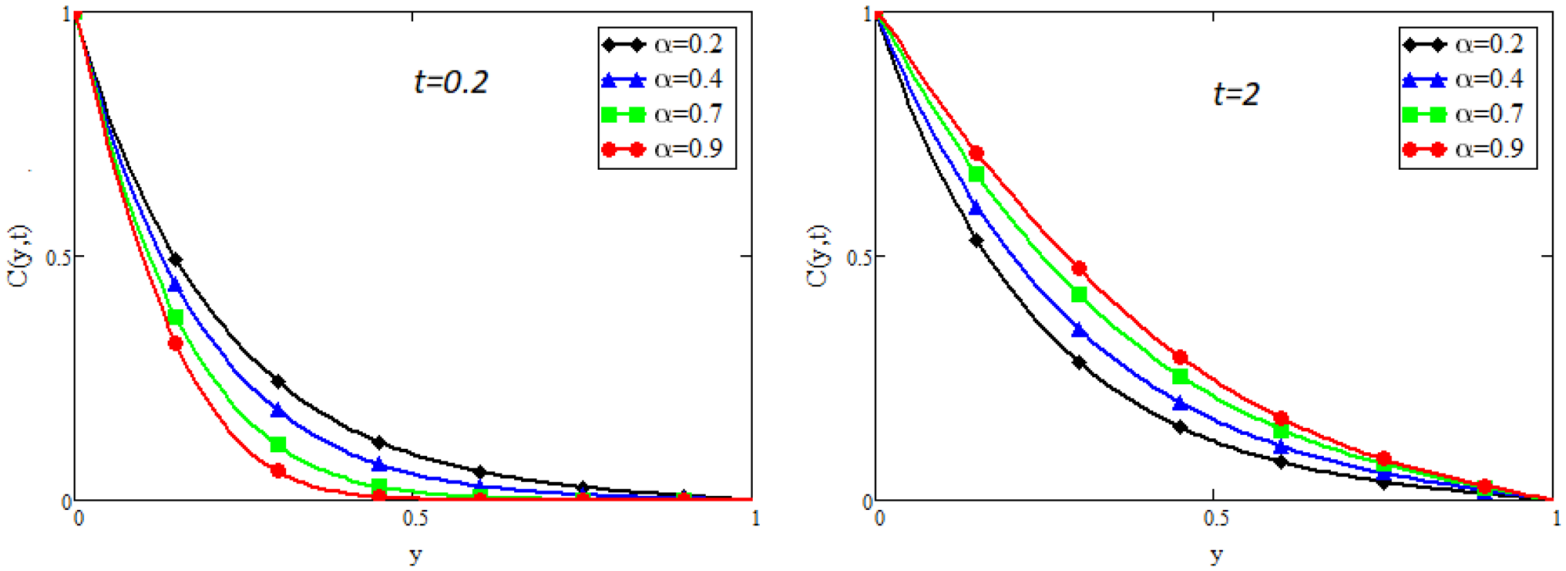
Impact of $\alpha$ on concentration distribution when $Sc=20$.

**Figure 12:**
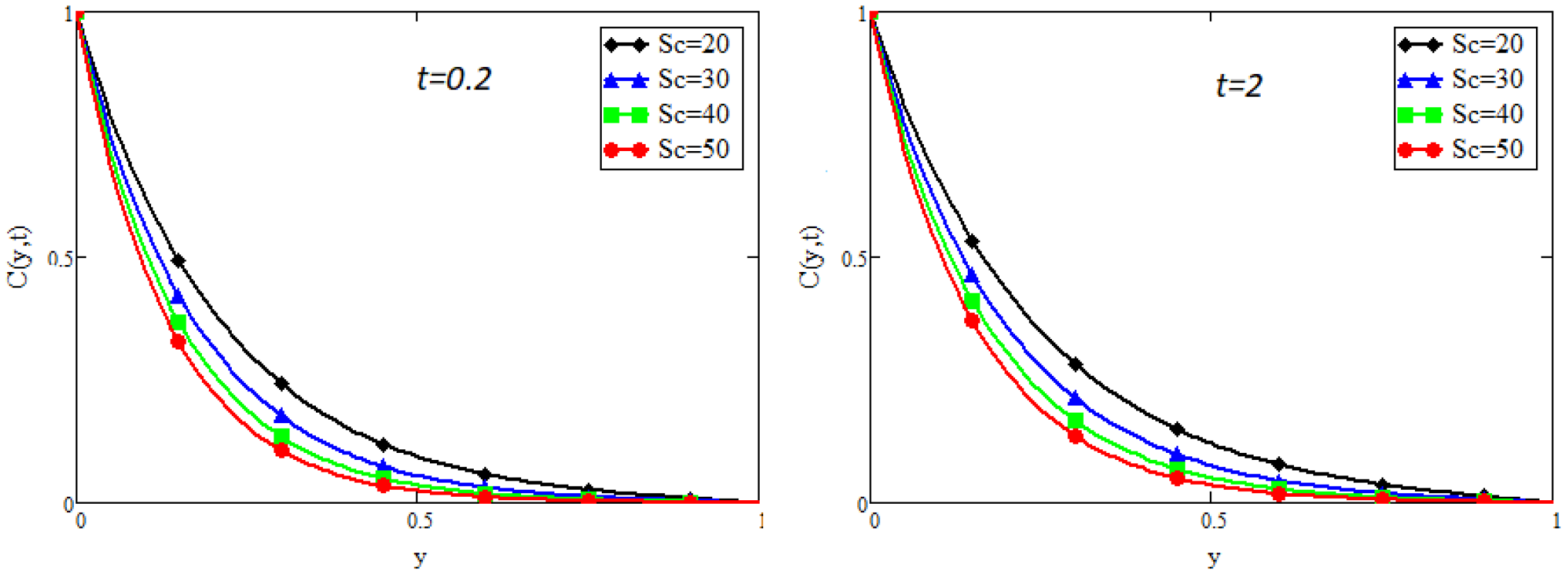
Impact of *Sc* on concentration distribution.

In this study, the unsteady flow of couple stress Casson fluid in a channel is investigated. By using Fick’s and Fourier’s laws a fractional model is developed. The closed form solutions are obtained by using the joint application of Laplace and Fourier finite sine transforms. The numerical values for the skin friction, Nusselt and Sherwood number of the flow are calculated and shown in tabular form. To show the effects of various embedded parameters on the velocity temperature and concentration distributions, Figs. 2, 3, 4, 5, 6, 7, 8, 9, 10, 11, 12 are drawn.

**Table 1:** Skin friction of the couple stress Casson fluid at the plate $y=0$ for $\alpha =0.2$.

*t*	$\beta$	*Sc*	*Pr*	$\lambda$	$G_m$	$G_r$	$S_{f}$
0.2	2	20	50	1	1.5	1.5	22.96
0.2	$\varvec{2.1}$	20	50	1	2	1.5	26.876
0.2	2	$\varvec{21}$	50	1	1.5	1.5	23.045
0.2	2	20	$\varvec{51}$	1	1.5	1.5	23.115
0.2	2	20	50	$\varvec{1.1}$	1.5	1.5	39.437
0.2	2	20	50	1	$\varvec{1.6}$	1.5	22.318
0.2	2	20	50	1	1.5	$\varvec{1.6}$	22.821

Significance values are in Bold.

**Table 2:** Nusselt number of the couple stress Casson fluid at the plate $y=0$ for $\alpha =0.2$.

*t*	*Pr*	*Nu*
2	20	3.87
2.1	20	3.851
2	**21**	3.965

Significance values are in Bold.

**Table 3:** Sherwood number of the couple stress Casson fluid at the plate $y=0$ for $\alpha =0.2$.

*t*	*Sc*	*Nu*
2	20	4.175
**2.1**	20	4.165
2	**21**	4.278

Significance values are in Bold.

Figure 2 shows the impact of fractional parameter $\alpha$ on the velocity distribution. From the figure, it is evident that unlike the classical model, multiple integral curves of velocity are obtained. These multiple integral curves may be best fitted with the experimental results or real data. Figures 3 and 4 show the influence of thermal Grashof number *Gr* and mass Grashof number *Gm* on the velocity of couple stress Casson fluid. These Portrayed figures exhibit that velocity is the increasing function of these numbers. It is physically true because the increase in *Gr* and *Gm* increase the buoyancy forces that decrease the viscosity of the fluid and hence increase in velocity occurs. In Fig. 5, the effect of the Prandtl number on the velocity. As the Prandtl number is the ratio of viscous forces to thermal forces. Thus rise in Prandtl number means that the viscous forces become dominant over thermal forces, which causes to decrease the velocity. Figure 6 captured the impact of Schmidt number. As Schmidt number is the ratio of viscous forces to mass diffusion, therefore an increase in Schmidt number causes to increase viscous forces and decrease mass diffusion and thus, the velocity of the fluid decreases. Figure 7 captured the impact of Casson parameter $\beta$ on the velocity profile. It is clear from the graph that by increasing the value of Casson parameter $\beta$, the velocity of the flow decreases. The physics of this behavior is, by increasing the value of Casson parameter increases the viscous forces that producing resistance to the flow and retards the flow. The impact couple stress parameter $\lambda$ on the velocity is shown in Fig. 8, which shows that velocity profile is the decreasing function of couple stress parameter $\lambda$. The physics of this behavior is, by increasing the Couple stress parameter, the viscosity increases which reduce the fluid velocity.

Figure 9 captures the temperature profile for different values of fractional parameter $\alpha$. From the figure, it is can be easily seen that it has dual effect. For small time i.e. $(t=0.2)$ the effect is quite opposite to that for large time $t=2$. The impact of Prandtl number *Pr* on temperature profile is displayed in Fig. 10. It shows that the temperature profile is the decreasing function of *Pr*. Physically it is true because Prandtl number is the ratio of viscosity to the thermal diffusivity, so by increasing the Prandtl number the viscosity of the fluid also increases and thus rise in temperature occurs. Concentration profile for different values of fractional parameter $\alpha$ is presented in Fig. 11. Here the behavior of concentration distribution is similar to temperature distribution. Figure 12 exhibited concentration distribution for different values of Schmidt number *Sc* which shows that concentration distribution behavior is unchanged for small time as well for large time. As concentration depends on viscosity, an increase in Schmidt number increase viscosity, and thus concentration profile increases.

Table 1 shows the variation in skin friction due to the change in values of different parameters. Skin friction is very important in different field of engineering specially civil engineering.The increasing values of the Casson parameter $\beta$, increases the viscous forces and hence the skin friction increases. Also if we increase the Schmidt number *Sc*, the viscous forces increases that causes to increase the skin friction. As Prandtl number *Pr* is proportional to viscous forces, so increase in *Pr* increases the viscous forces that causes to increases the skin friction. By increasing the couple stress parameter $\lambda$, the viscosity of the fluid increases which increases the skin friction shown in the Table 1. Finally, by increasing thermal Grashof number $G_r$ and mass Grashof number $G_m$, the skin friction also decreases. The physics of this behavior is, by increasing in $G_r$ and $G_m$ the buoyancy forces increases that decreases the viscosity and hence the skin friction decreases. Table 2 shows Nusselt number. As Prandtl number *Pr* is the ratio of momentum diffusivity to thermal diffusivity, so increase in *Pr* increase the momentum diffusion which reduces the thermal boundary layer thickness that decreases the Nusselt numbes. Table 3 shows Sherwood number. As we increase the time , the Sherwood number decrease and by increasing Schmidt number, the sherwood number increases. This physics is that, Shmidt number is the ratio of viscous forces to the mass diffusion. Increasing Schmidt number increases viscous forces and decreases the mass diffusion and hence the Sherwood number increases.

## Conclusion

This manuscript deals with the modern approach of Fick’s and Fourier’s laws to transform the classical model to time-fractional model using the definition of Caputo. The integral transforms (Laplace and Fourier ) are used to find exact solutions. The impact of various embedded parameters on velocity, temperature, and concentration distributions is presented graphically and discussed physically. The concluding points of the current work are listed below:

1. The Fick’s and Fourier’s laws are used to transform the time derivative to time-fractional model.
2. The fractional models are more realistic which provides many solutions. These solutions may be best fit to the real data.
3. The couple stress parameter $\lambda$ retards the velocity for its increasing values.
4. The behavioral changes in the velocity of couple stress Casson fluid is positive with an increasing number of Grashof $G_r$ and $G_m$, while the increasing values of *Pr*, *Sc*, and $\beta$ bring an opposite behavior to that of $G_m$ and $G_r$.
5. The effect of various parameters on the skin friction is quite opposite to that of velocity which is an good agreement with the definition of skin friction.
